# Regulator of calcineurin 3 as a novel predictor of diagnosis and prognosis in pan-cancer

**DOI:** 10.3325/cmj.2024.65.356

**Published:** 2024-08

**Authors:** Yuan Liang, Wenjuan Diao, Xuefen Yang, Yue Tao, Longnian Hong, Wenle Li

**Affiliations:** 1 Department of Gynecology, The First Hospital of Qiqihar, Qiqihar, Heilongjiang, China; 2 Department of Gynecology, Affiliated Qiqihar Hospital, Southern Medical University, Qiqihar, Heilongjiang, China; 3 Department of Gynecology, Affiliated Hospital of Guangdong Medical University, Zhanjiang, Guangdong, China

## Abstract

**Aim:**

To assess diagnostic and prognostic value of regulator of calcineurin 3 (RCAN3) in various malignancies.

**Methods:**

RCAN3 expression levels were assessed across pan-cancer data sets including various molecular and immune subtypes. Receiver operating characteristic (ROC) and Kaplan-Meier curves were employed to determine the diagnostic and prognostic value of RCAN3 in pan-cancer, respectively. Enrichment analyses were used to identify RCAN3-associated terms and pathways. A special focus was placed on cervical squamous cell carcinoma and endocervical adenocarcinoma cervical cancer (CESC); we assessed the prognostic value of RCAN expression within distinct clinical subgroups and its effect on m6A modifications and immune infiltration.

**Results:**

RCAN3 expression varied not only in different cancer types but also in different molecular and immune subtypes of cancers. RCAN3 displayed high accuracy in diagnosing and predicting cancers, and RCAN3 expression level was associated with the prognosis of certain cancers. CESC patients with a high RCAN3 level had a worse overall survival, disease-specific survival, and progression-free interval. RCAN3 expression was related to multiple m6A modifier genes and immune cells.

**Conclusion:**

In general, RCAN3 can serve as a novel biomarker for the diagnosis and prognosis in pan-cancer, especially in CESC. It may represent a promising molecular target for developing new treatments. However, our analysis is limited to bioinformatic predictions, and further biological experiments are necessary to verify our results.

The regulator of calcineurin (*RCAN*) gene family encompasses three functional subfamily members: *RCAN1* (1), *RCAN2* (2), and *RCAN3* (3). The *RCAN3* consists of five exons and is located on chromosome 1 (1p36.11), encoding a protein of 241 amino acids (4). RCAN3 acts by inhibiting the phosphatase activity of calcineurin (CaN); this interaction occurs primarily through the Capicua (CIC) motif of RCAN3. CaN is a calcium-dependent serine/threonine protein phosphatase, and its activity regulation plays an important role in many biological processes (5-13). The activity of CaN is influenced by calmodulin and the CaN regulatory domain (RD). When calmodulin is combined with the calmodulin identification area (calmodulin binding region, CaMBR) within the CaN RD, it can be driven to fold and activate CaN. However, RCAN3 is able to inhibit the full activation process, preventing the C-terminal region of CaMBR (“distal helix”) from exhibiting an alpha-helix fold. By inhibiting the activity of CaN, RCAN3 is involved in the regulation of nuclear factor of activated T cells (NFAT) signals. NFAT is a CaN-dependent endogenous regulator, and both its nuclear translocation and regulation of the NFAT downstream pathway are affected by RCAN3. Through this, RCAN3 plays an active role in improving autoimmune diseases. RCAN3 defects have been found to lead to decreased cell proliferation, increased apoptosis, and abnormal mitochondrial ultrastructure (14). Notably, RCAN3 and its derived peptide may impede breast cancer progression by inhibiting the CaN-NFATc signaling pathway (15). RCAN3 expression is increased in cervical cancer tissues and correlates with adverse outcomes (16). Furthermore, RCAN3 knockdown inhibits the proliferation, invasion, and migration of cervical cancer cells (17). RCAN3 is unique in its ability to directly affect the activity of CaN and, in turn, the concentration of calcium ions within cells, which is essential for cell survival and death.

Due to the central role of calcium signaling in cellular physiological and pathological processes, abnormal expression or dysfunction of RCAN3 may lead to changes in cellular behavior that contribute to the onset and development of cancer. Therefore, a better understanding of RCAN3-related mechanisms could help in the development of new anti- treatment strategies, especially in cancer types that cannot be effectively treated. At present, RCAN3 can be used as a reliable marker for three cancer types (breast cancer, endometrial adenocarcinoma, and bladder cancer); however, its role in pan-cancer is less well-studied. Previous cancer studies have focused more on gene mutations, cell cycle regulation, and signaling pathways, and research on RCAN3 may provide a new perspective on how to prevent cancer progression by regulating calcium signaling pathways. Therefore, this research aimed to determine the potential of RCAN3 as a diagnostic and prognostic biomarker in pan-cancer studies and as a molecular target for addressing cervical squamous cell carcinoma and endocervical adenocarcinoma cervical cancer (CESC).

## MATERIALS AND METHODS

### Data sources

The RNA-seq data related to 33 tumor types and normal tissues of 15 776 samples were obtained from The Cancer Genome Atlas (TCGA) database and the Genotype-Tissue Expression (GTEx) database, respectively. The GTEx database collects gene expression and genotype data of >50 human tissue samples, allowing the study of the association between gene expression and genetic variation. TCGA is a comprehensive human cancer database containing data on >30 cancer types, >30 000 tumor samples, and >20 000 accounts of gene expression information. The analysis was conducted in July 2022.

### Regulator of calcineurin 3 expression in immune and molecular subtypes of cancers

RCAN3 expression in different immune and molecular subtypes in pan-cancer was assessed through The Tumor and Immune System Interaction Database (TISIDB) (http://cis.hku.hk/TISIDB), an online portal for investigation of tumor-immune interactions that collects multiple human cancer data types (18)

### Diagnostic value analysis

The diagnostic performance of RCAN3 was assessed with the receiver operating characteristic (ROC) curve and the resulting area under the curve (AUC); AUC>0.7 was considered to indicate good diagnostic discriminatory property.

### Survival analysis

The Kaplan-Meier (KM) survival curve (Cox method) was used to evaluate the effects of RCAN3 on overall survival (OS), disease-specific survival (DSS), and progression-free interval (PFI) in all patients. We further assessed the prognosis (OS, DSS, and PFI) in different clinical subgroups of CESC.

### Enrichment analyses

Fifty RCAN3-associated proteins were identified using the Search Tool for the Retrieval of Interacting Genes/Proteins (STRING) database (https://string-db.org/), with a medium confidence level (requiring a minimum interaction score >0.400) as the selection criterion. Based on the identified proteins, gene ontology (GO) and Kyoto Encyclopedia of Genes and Genomes (KEGG) enrichment analyses were conducted. In the KEGG enrichment analysis, the ID of a differentially expressed gene or protein was mapped to the corresponding entry in the KEGG database to determine the pathway to which the gene or protein belonged.

### Associations between regulator of calcineurin 3 expression and m6A modification in cervical cancer

The analysis involved examining the correlation between RCAN3 expression and the expression of m6A-related genes (*METTL3, YTHDC1, YTHDC2, METTL14, RBM15, RBM15B, IGF2BP1, IGF2BP2, IGF2BP3, VIRMA, WTAP, YTHDF1, YTHDF2, YTHDF3, ZC3H13, HNRNPA2B1, HNRNPC, RBMX, FTO,* and *ALKBH5*) (19) in CESC.

### Correlations between regulator of calcineurin 3 expression and immune infiltration

To calculate immune infiltration, we used markers provided by Bindea et al (20) for 24 immune cell types based on the ssGSEA algorithm provided by the R package – Gene Set Variation Analysis (GSVA; 1.46.0) (21).

### Statistical analysis

Statistical analyses were conducted with the R software (v. 3.6.3). Differences in RCAN3 expression between cancer and normal tissues were assessed with the Wilcoxon rank sum test. Using Log2(value +1), we normalized the data to avoid the effects of maximum, negative, and extreme values. For survival analysis and visualization, the R packages “survival (v3.2.1)” and “survminer (v3.3.3)” were used. For gene ontology enrichment analysis, ClusterProfiler (R package) was used. Gene ontology and KEGG enrichment analyses were performed using the “ggplot2” and “clusterProfiler” R packages. Differential gene expression analysis was conducted using “DESeq2”, and GSEA was conducted using “clusterProfiler.” Differences in the expression of m6A-related genes between groups with high and low RCAN3 expression were assessed with the Spearman correlation coefficient and the Wilcoxon rank sum test. The matrix and immunity scores were calculated using the R-package estimate (1.0.13) (22). The “GSVA” and “estimate” package were used to assess the immune cell infiltration and immune score, respectively. For all analyses, the level of significance was set at *P* < 0.05.

## Results

### Regulator of calcineurin 3 expression in pan-cancer

**Figure 1:**
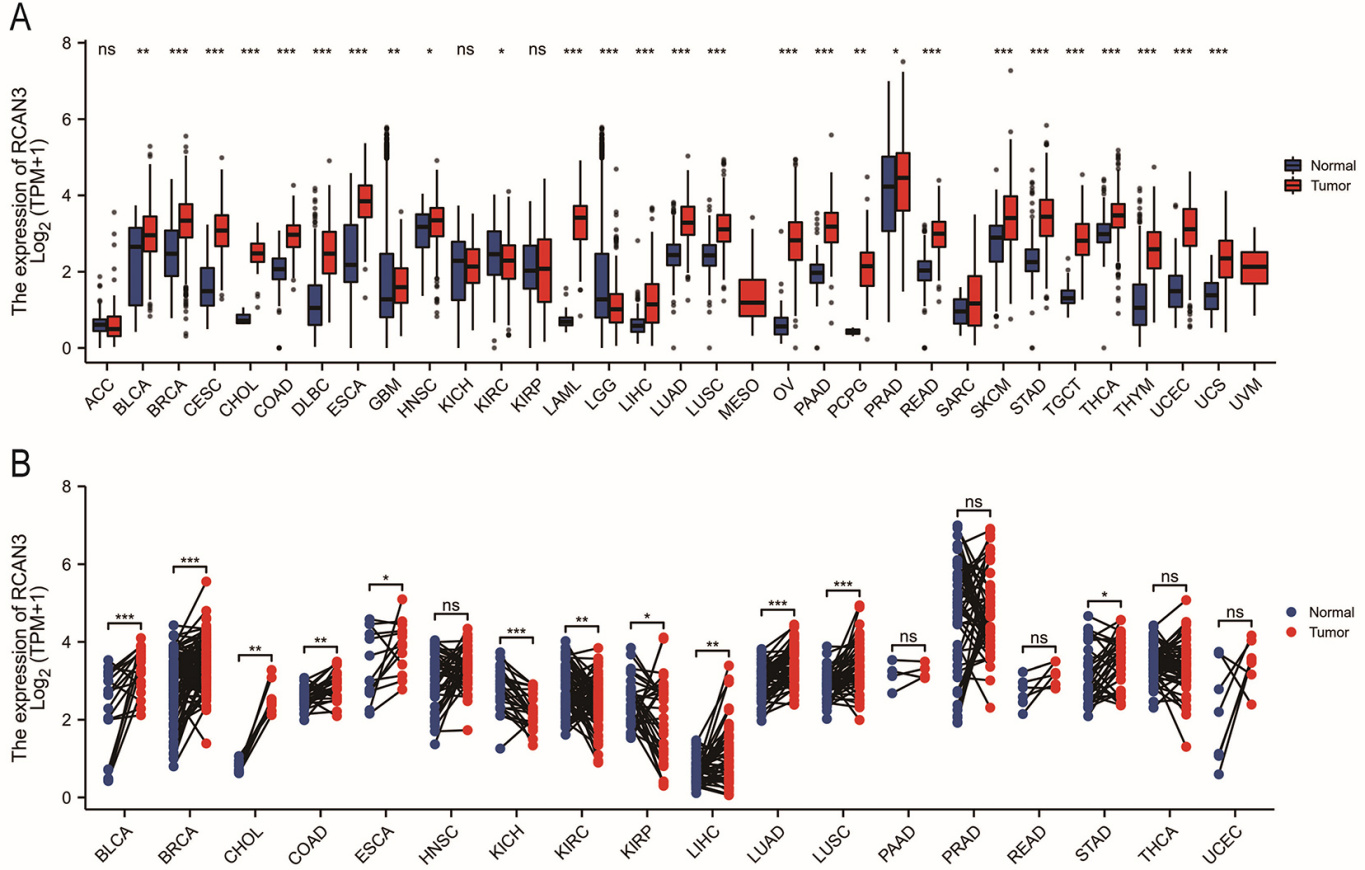
(**A**) Regulator of calcineurin 3 (RCAN3) expression in The Cancer Genome Atlas (TCGA) tumors and genotype-tissue expression in normal tissues; (**B**) RCAN3 expression in paired normal and tumor tissues in TCGA (*P < 0.05, **P < 0.01, ***P < 0.001). Abbreviations: bladder urothelial carcinoma (BLCA); breast invasive carcinoma (BRCA); cervical squamous cell carcinoma and endocervical adenocarcinoma cervical cancer (CESC); cholangiocarcinoma (CHOL); colon adenocarcinoma (COAD); diffuse large B-cell lymphoma (DLBC); esophageal carcinoma (ESCA); glioblastoma (GBM); head and neck squamous cell carcinoma (HNSC); acute myeloid leukemia (LAML); liver hepatocellular carcinoma (LIHC); lung adenocarcinoma (LUAD); lung squamous cell carcinoma (LUSC); ovarian serous cystadenocarcinoma (OV); pancreatic adenocarcinoma (PAAD); pheochromocytoma and paraganglioma (PCPG); prostate adenocarcinoma (PRAD); rectum adenocarcinoma (READ); skin cutaneous melanoma (SKCM); stomach adenocarcinoma (STAD); testicular germ cell tumors (TGCT); thyroid carcinoma (THCA); thymoma (THYM); kidney renal clear cell carcinoma (KIRC); lower-grade glioma (LGG); kidney chromophobe (KICH); kidney renal papillary cell carcinoma (KIRP); uterine corpus endometrial carcinoma (UCEC).

RCAN3 was significantly upregulated in various cancers, including bladder urothelial carcinoma (BLCA), breast invasive carcinoma (BRCA), CESC, cholangiocarcinoma (CHOL), colon adenocarcinoma (COAD), diffuse large B-cell lymphoma (DLBC), esophageal carcinoma (ESCA), glioblastoma (GBM), head and neck squamous cell carcinoma (HNSC), acute myeloid leukemia (LAML), liver hepatocellular carcinoma (LIHC), lung adenocarcinoma (LUAD), lung squamous cell carcinoma (LUSC), ovarian serous cystadenocarcinoma (OV), pancreatic adenocarcinoma (PAAD), pheochromocytoma and paraganglioma (PCPG), prostate adenocarcinoma (PRAD), rectum adenocarcinoma (READ), skin cutaneous melanoma (SKCM), stomach adenocarcinoma (STAD), testicular germ cell tumors (TGCT), thyroid carcinoma (THCA) and thymoma (THYM). Conversely, RCAN3 was downregulated in kidney renal clear cell carcinoma (KIRC) and lower-grade glioma (LGG) (Figure 1A). Additionally, RCAN3 expression in paired normal and tumor tissues from TCGA database was consistently upregulated in several cancers, such as BLCA, BRCA, CHOL, COAD, ESCA, LIHC, LUAD, LUSC, and STAD; in contrast, it was downregulated in kidney chromophobe (KICH), KIRC, and kidney renal papillary cell carcinoma (KIRP; Figure 1B).

### Regulator of calcineurin 3 expression in immune and molecular subtypes of cancer

**Figure 2:**
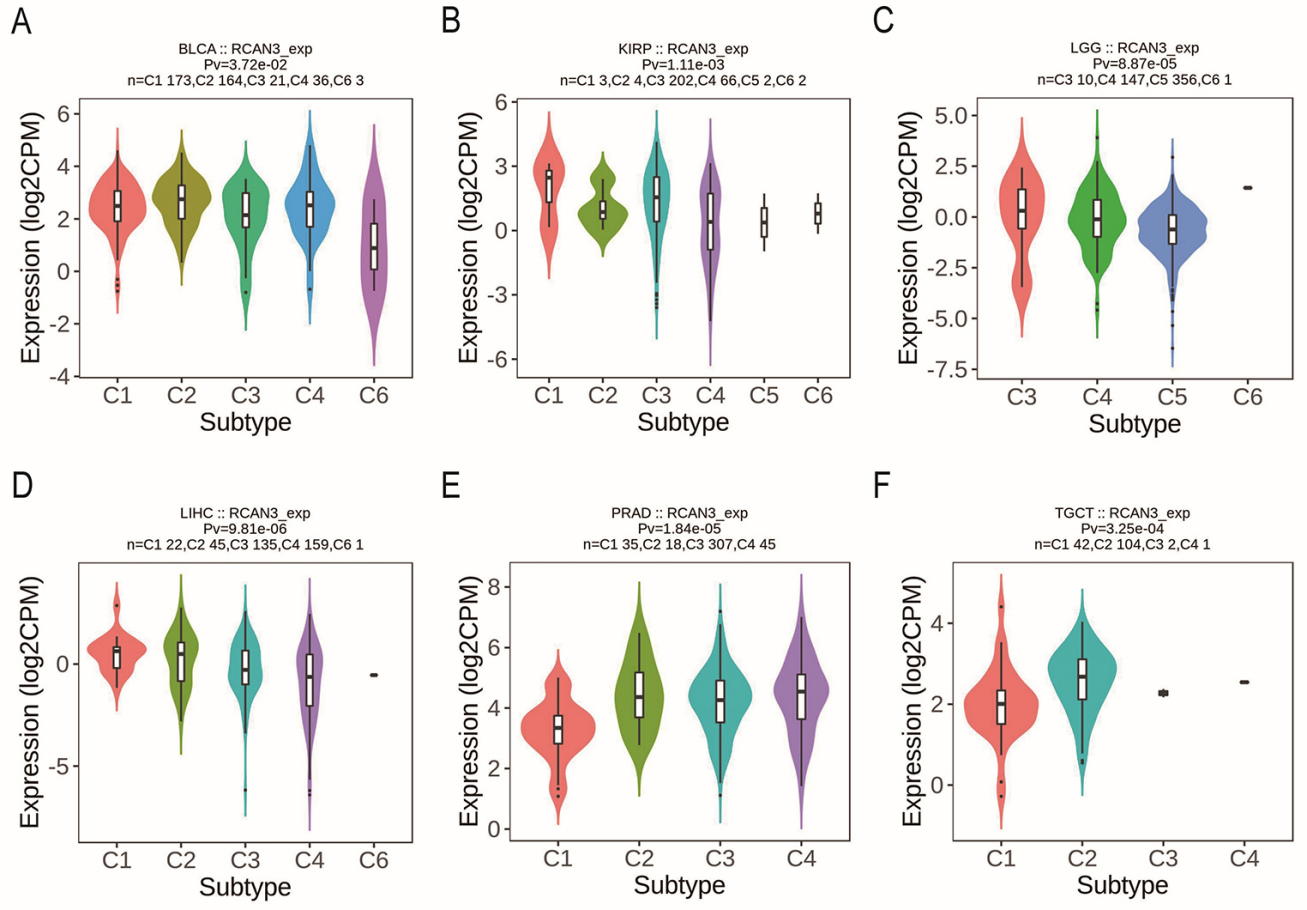
Associations between regulator of calcineurin 3 (RCAN3) expression and immune subtypes across The Cancer Genome Atlas tumors (Kruskal-Wallis H test). (**A**) BLCA; (**B**) KIRP; (**C**) LGG; (**D**) LIHC; (**E**) PRAD; (**F**) TGCT. C1 – wound healing; C2 – IFN-gamma dominant, C3 – inflammatory; C4 – lymphocyte depleted; C5 – immunologically quiet, and C6 – TGF-b dominant. See the legend to Figure 1 for abbreviations.

**Figure 3:**
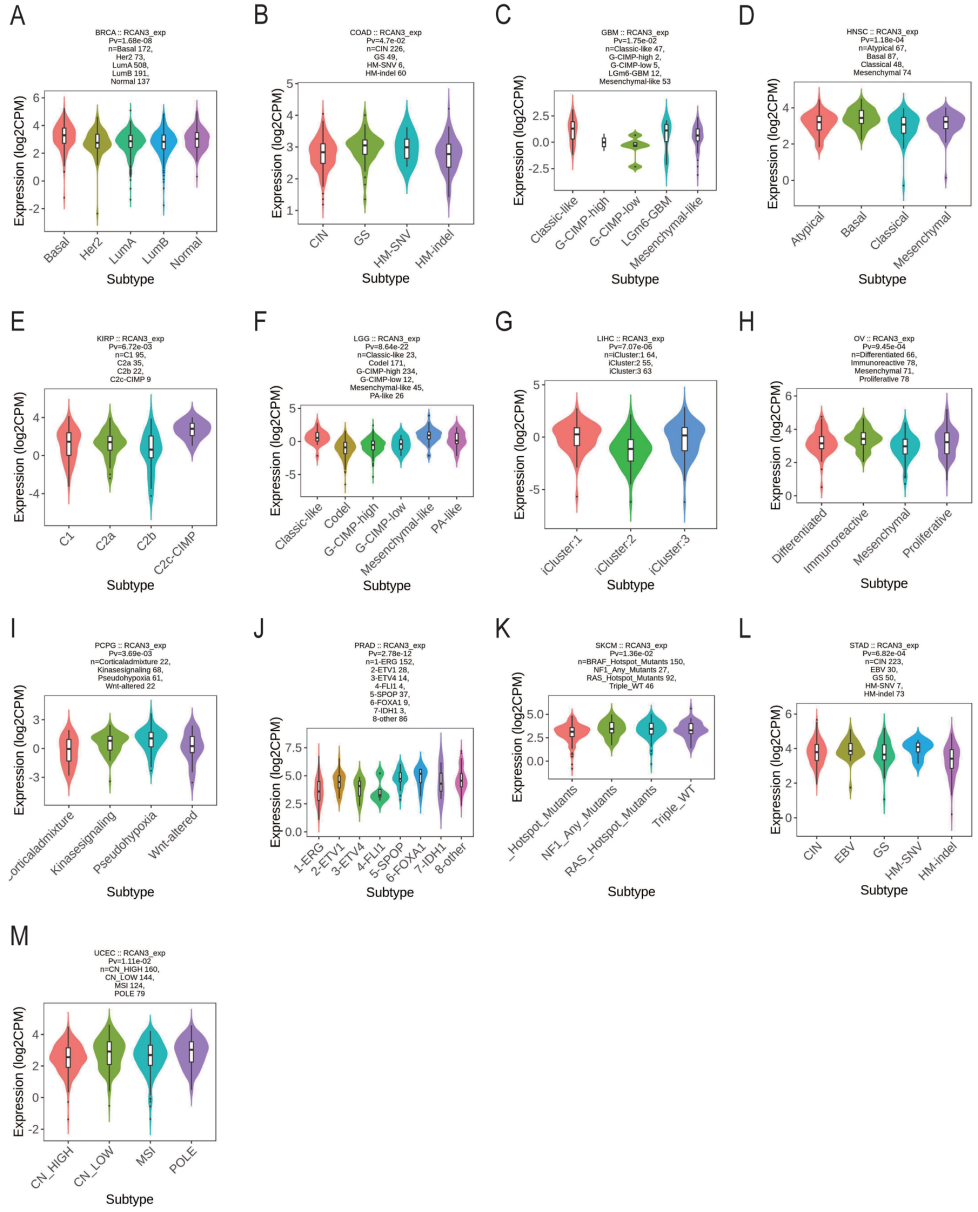
Associations between regulator of calcineurin 3 (RCAN3) expression and molecular subtypes across The Cancer Genome Atlas (TCGA) tumors (Kruskal-Wallis H test). (**A**) BRCA; (**B**) COAD; (**C**) GBM; (**D**) HNSC; (**E**) KIRP; (**F**) LGG; (**G**) LIHC; (**H**) OV; (**I**) PCPG; (**J**) PRAD; (**K**) SKCM; (**L**) STAD; (**M**) UCEC. See the legend to Figure 1 for abbreviations.

RCAN3 expression was associated with different immune subtypes (C1: wound healing, C2: IFN-gamma dominant, C3: inflammatory, C4: lymphocyte depleted, C5: immunologically quiet, and C6: TGF-b dominant) in BLCA, KIRP, LGG, LIHC, PRAD, and TGCT (Figure 2). Additionally, RCAN3 expression exhibited differential expression across molecular subtypes in cancers, including BRCA, COAD, GBM, HNSC, KIRP, LGG, LIHC, OV, PCPG, PRAD, SKCM, STAD, and uterine corpus endometrial carcinoma (UCEC; Figure 3).

### Diagnostic value of regulator of calcineurin 3 in pan-cancer

**Figure 4:**
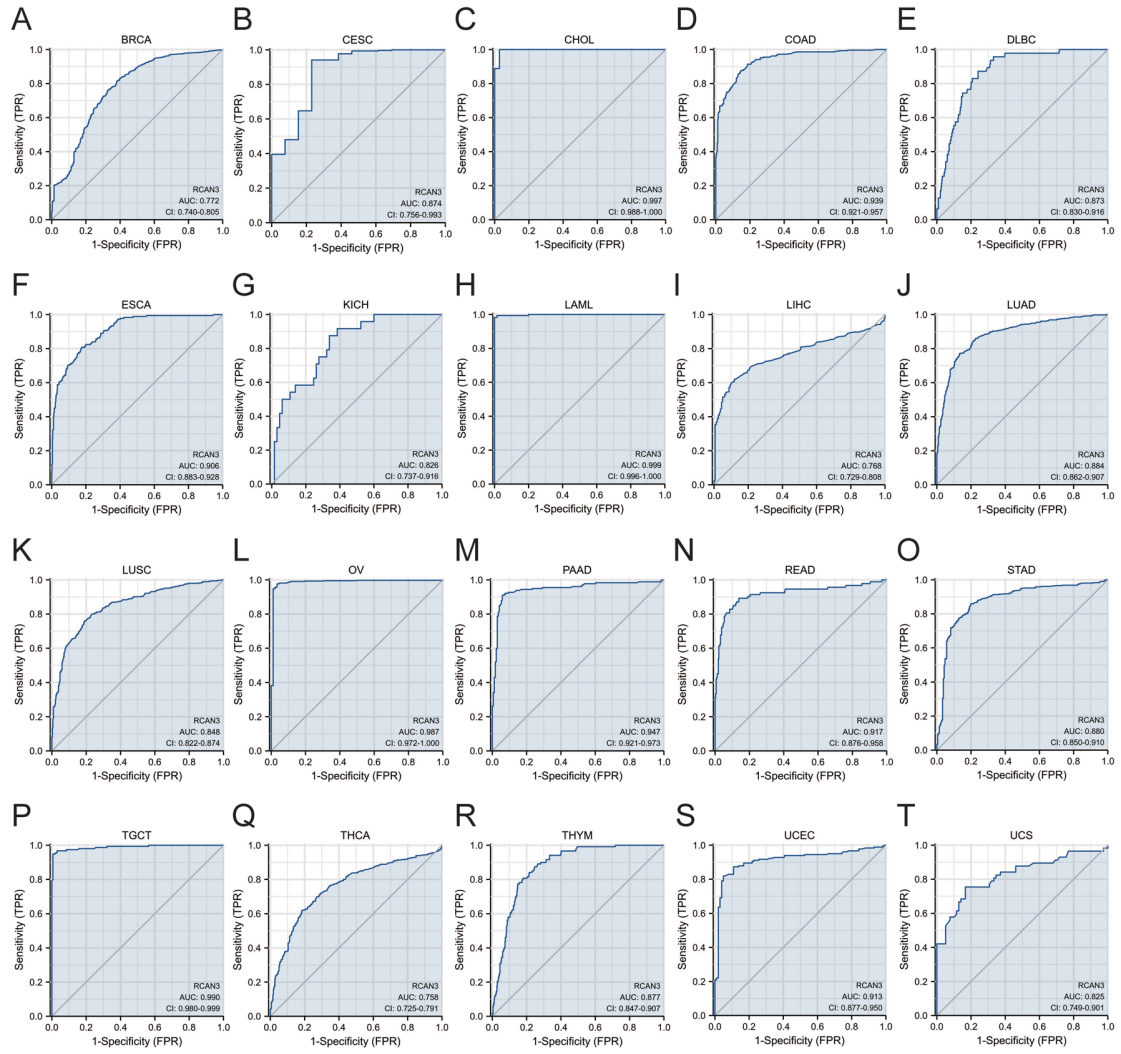
The receiver operating characteristic (ROC) curve analysis of regulator of calcineurin 3 (RCAN3) in pan-cancer. (**A**) BRCA; (**B**) CESC; (**C**) CHOL; (**D**) COAD; (**E**) DLBC; (**F**) ESCA; (**G**) KICH; (**H**) LAML; (**I**) LIHC; (**J**) LUAD; (**K**) LUSC; (**L**) OV; (**M**) PAAD; (**N**) READ; (**O**) STAD; (**P**) TGCT; (**Q**) THCA; (**R**) THYM; (**S**) UCEC; (**T**) UCS. See the legend to Figure 1 for abbreviations.

RCAN3 exhibited favorable diagnostic performance (AUC>0.7) for predicting 20 cancer types, including BRCA (AUC = 0.772), CESC (AUC = 0.874), CHOL (AUC = 0.997), COAD (AUC = 0.939), DLBC (AUC = 0.873), ESCA (AUC = 0.906), KICH (AUC = 0.826), LAML (AUC = 0.999), LIHC (AUC = 0.768), LUAD (AUC = 0.884), LUSC (AUC = 0.848), OV (AUC = 0.987), PAAD (AUC = 0.947), READ (AUC = 0.917), STAD (AUC = 0.880), TGCT (AUC = 0.990), THCA (AUC = 0.758), THYM (AUC = 0.877), UCEC (AUC = 0.913), and uterine carcinosarcoma (AUC = 0.825). Notably, RCAN3 displayed high diagnostic accuracy (AUC>0.9) in CHOL, COAD, ESCA, LAML, OV, PAAD, READ, TGCT, and UCEC (Figure 4).

### Prognostic value of RCAN3 in cancers

**Figure 5:**
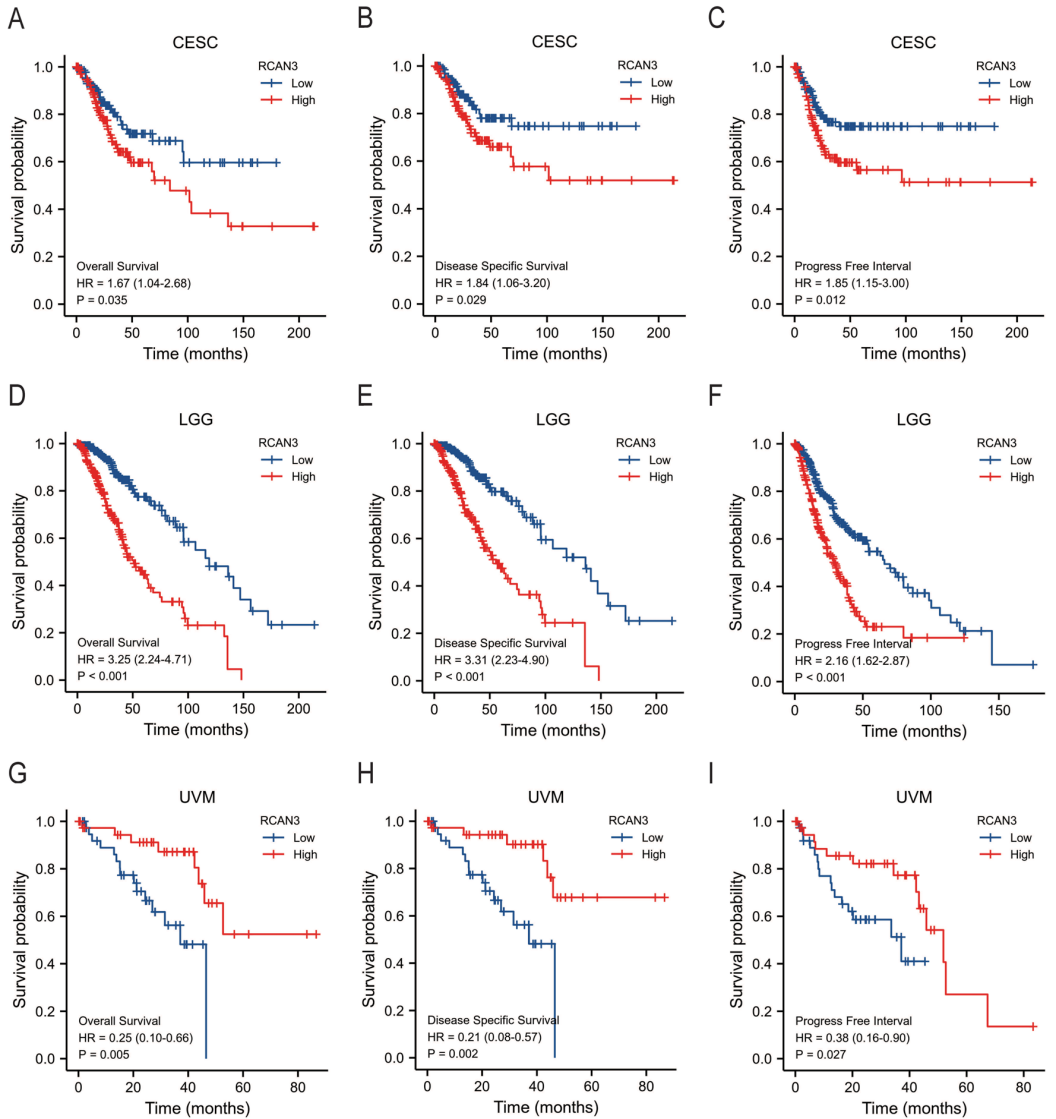
Survival analysis of regulator of calcineurin 3 (RCAN3) in cancers. (A-C) CESC; (D-F) LGG; (G-I) UVM. See the legend to Figure 1 for abbreviations.

**Figure 6:**
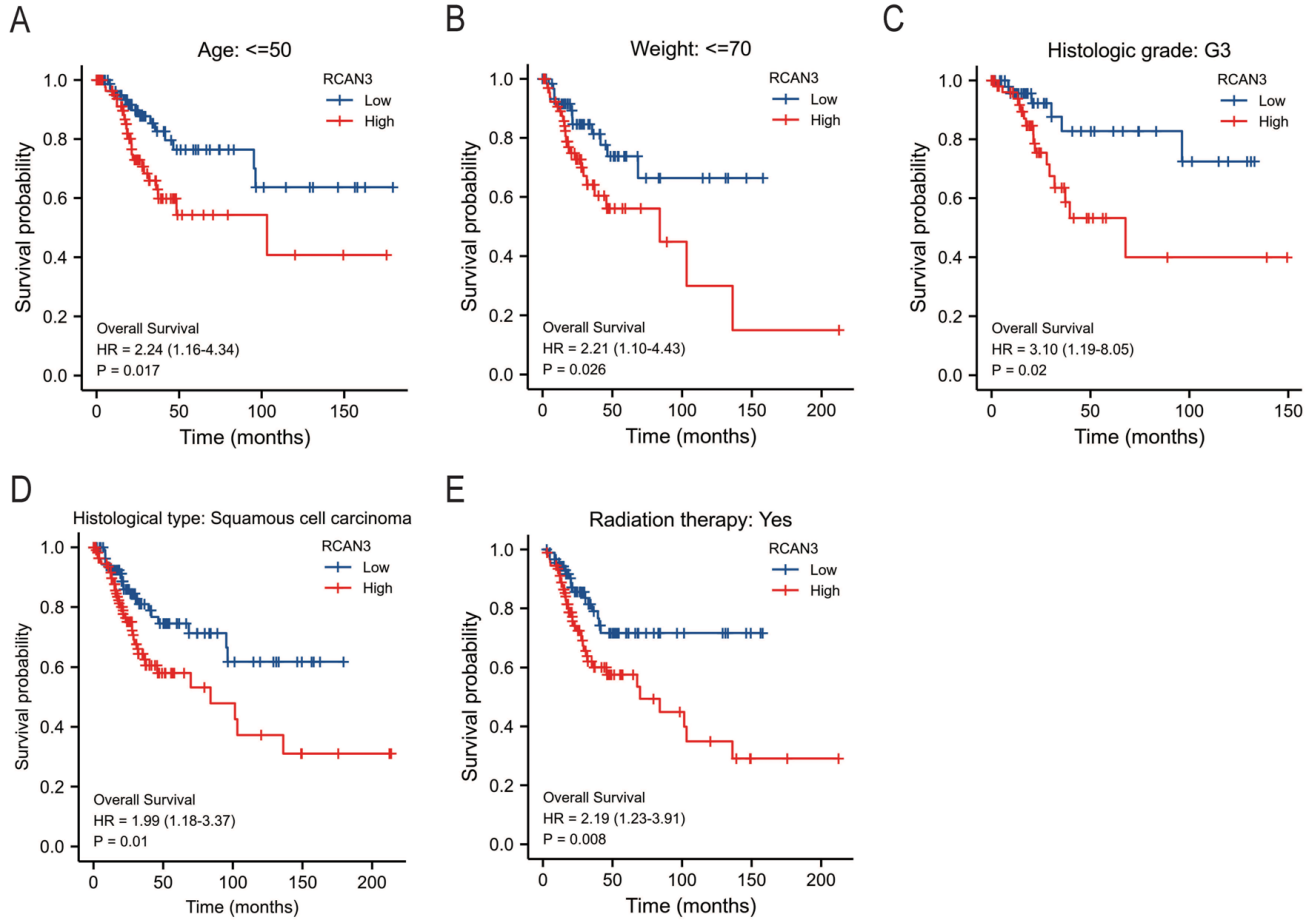
Overall survival analysis of regulator of calcineurin 3 (RCAN3) in clinical subgroups of cervical squamous cell carcinoma and endocervical adenocarcinoma (CESC) according to (**A**) age ≤50; (**B**) weight ≤70 kg; (**C**) histologic grade (G3); (**D**) histological type (squamous cell carcinoma); (**E**) radiation therapy (yes).

**Figure 7:**
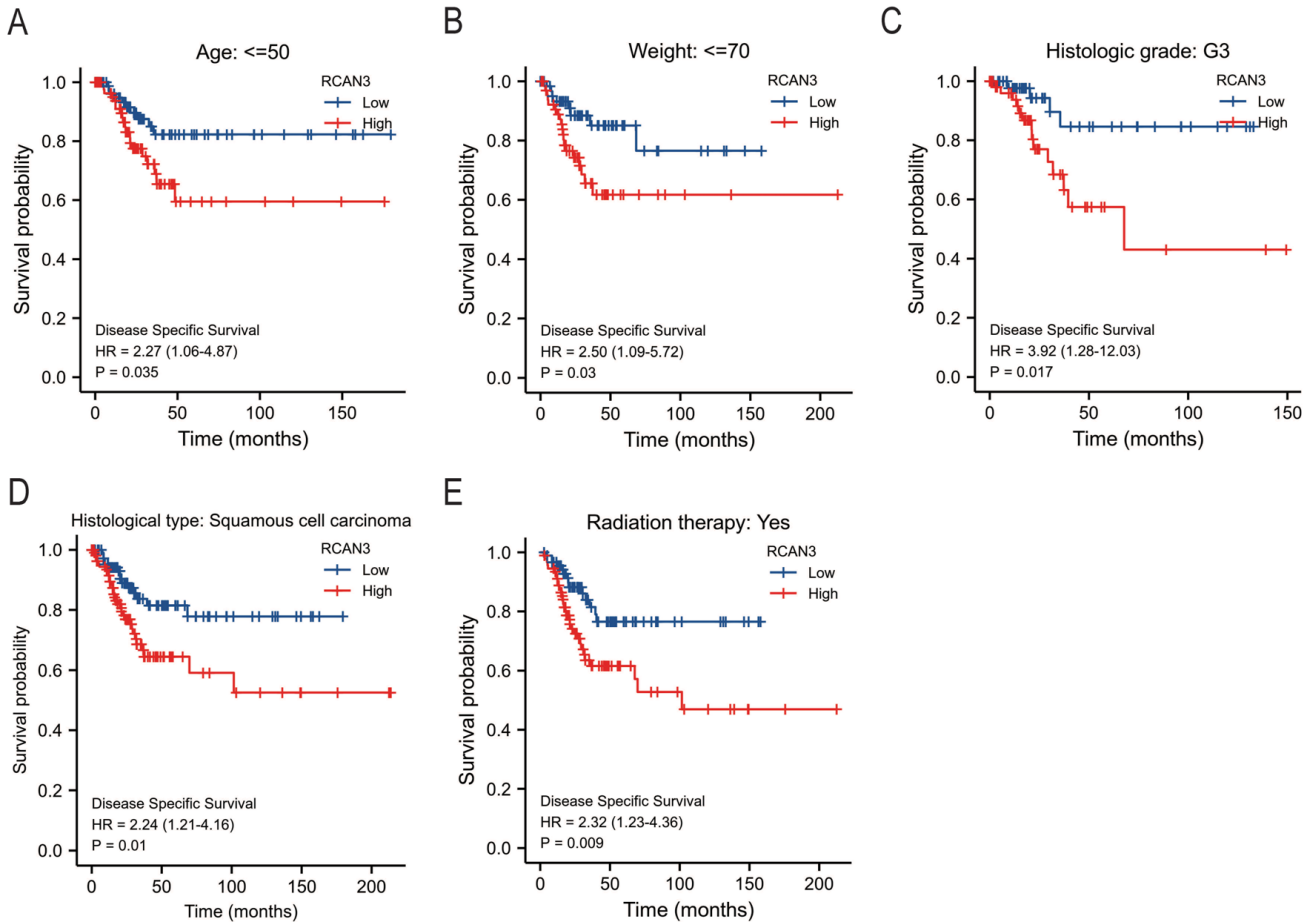
Disease-specific survival analysis of regulator of calcineurin 3 (RCAN3) in clinical subgroups of cervical squamous cell carcinoma and endocervical adenocarcinoma (CESC) according to (**A**) age ≤50; (**B**) weight ≤70; (**C**) histologic grade (G3); (**D**) histological type (squamous cell carcinoma); (**E**) radiation therapy (yes).

**Figure 8:**
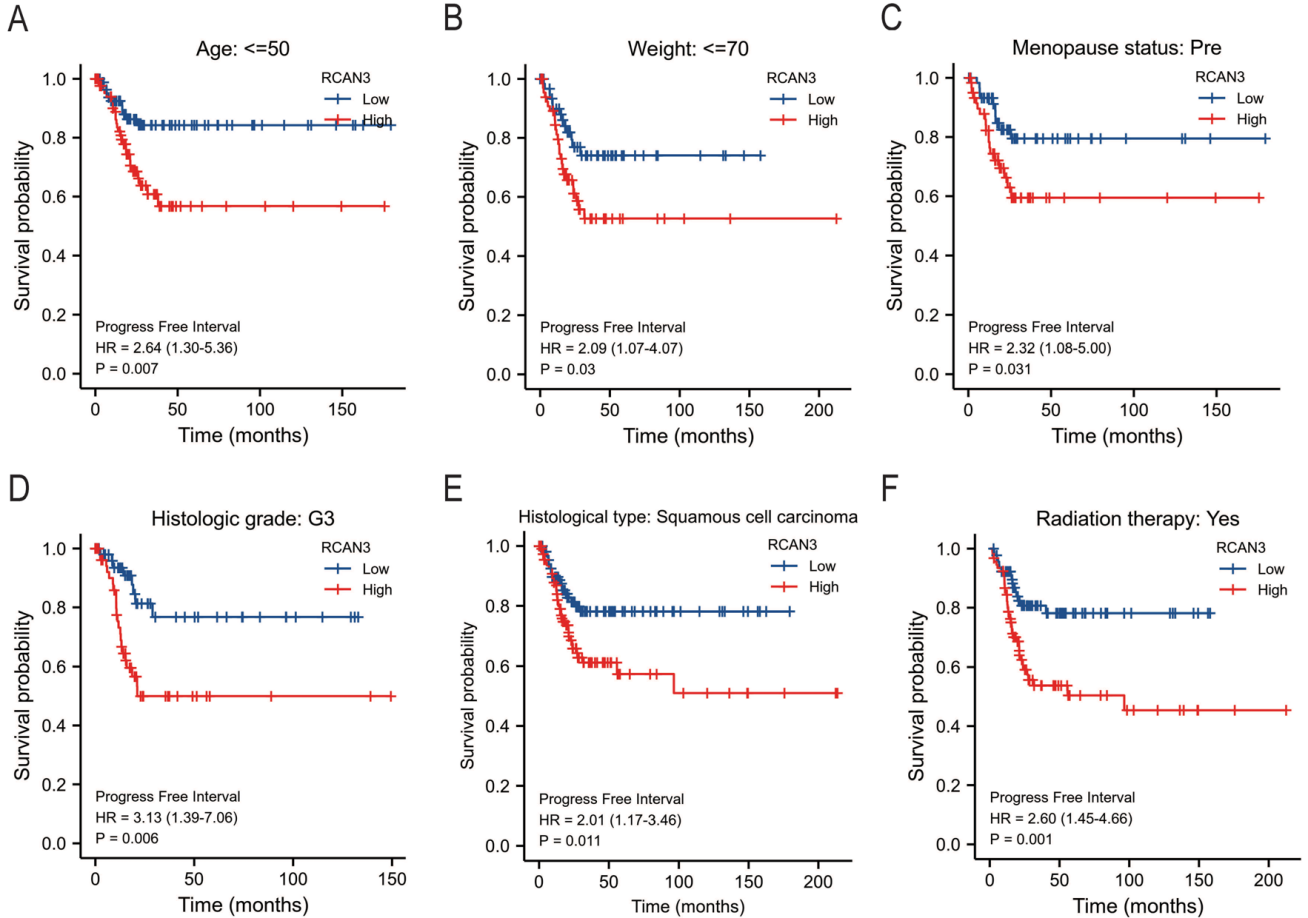
Progression-free interval analysis of regulator of calcineurin 3 (RCAN3) in clinical subgroups of cervical squamous cell carcinoma and endocervical adenocarcinoma (CESC) according to (**A**) age ≤50; (**B**) weight ≤70; (**C**) menopause status (pre); (**D**) histologic grade (G3); (**E**) histological type (squamous cell carcinoma); (**F**) radiation therapy (yes).

The KM survival curve analysis revealed the impact of RCAN3 expression on patient prognosis (OS, DSS, and PFI) in three cancer types: CESC, LGG, and uveal melanoma (UVM). High RCAN3 expression was associated with poor prognosis in CESC (OS *P* = 0.035, HR = 1.67; DSS *P* = 0.029, HR = 1.84; PFI *P* = 0.012, HR = 1.85; Figure 5A) and LGG (OS *P* < 0.001, HR = 3.25; DSS *P* < 0.001, HR = 3.31; PFI *P* < 0.001, HR = 2.16; Figure 5B). Surprisingly, in UVM, high RCAN3 expression was associated with a relatively good prognosis (OS *P* = 0.005, HR = 0.25; DSS *P* = 0.002, HR = 0.21; PFI *P* = 0.027, HR = 0.38; Figure 5C). Furthermore, a subgroup analysis in CESC indicated that RCAN3 overexpression was linked to poor OS and DSS in specific subgroups (age ≤50, weight ≤70, histologic grade G3, histological type squamous cell carcinoma, radiation therapy [yes]; Figure 6, 7). For PFI, high RCAN3 expression predicted poor prognosis in specific subgroups (age ≤50, weight ≤70, menopause status Pre, histologic grade G3, histological type squamous cell carcinoma, radiation therapy [yes]; Figure 8).

### Univariate and multivariate analyses in cervical cancer

**Figure 9:**
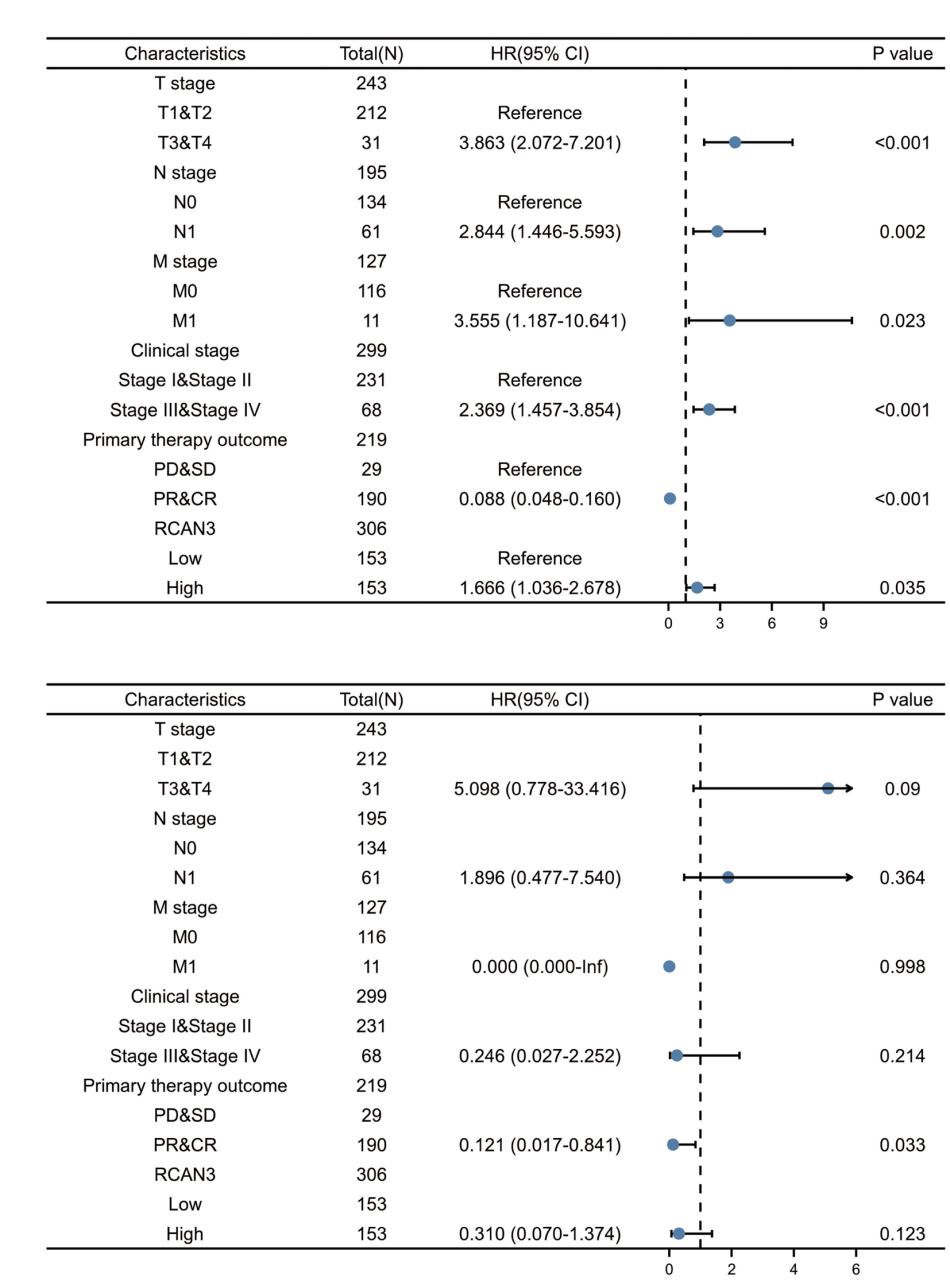
Univariate (**A**) and multivariate (**B**) Cox regression analyses of clinical features of cervical squamous cell carcinoma and endocervical adenocarcinoma (CESC) associated with overall survival. Abbreviations: PD – progressive disease; SD – stable disease; PR – partial response CR – complete response.

Univariate and multivariable analyses for OS in CESC identified the T stage, N stage, M stage, clinical stage, primary therapy outcome and RCAN3 as independent prognostic factors (Figure 9A). In the multivariate Cox model, the primary therapy outcome was significantly correlated with OS in patients with CESC (Figure 9B).

### Enrichment analyses

**Figure 10:**
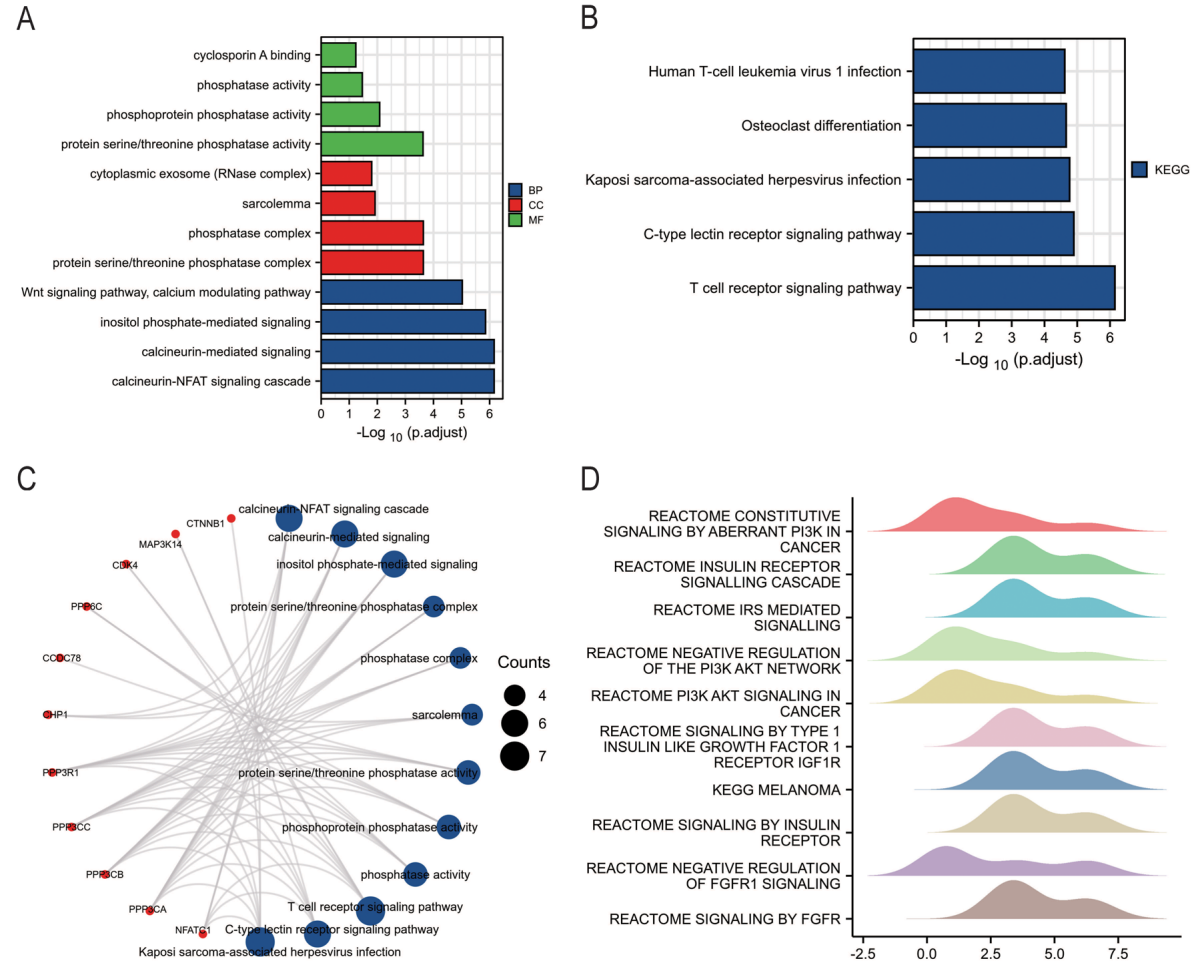
Enrichment analyses of regulator of calcineurin 3 (RCAN3). (**A-C**) gene ontology (GO) and Kyoto Encyclopedia of Genes and Genomes (KEGG) analyses of RCAN3-related genes; (**D**) gene set enrichment analysis (GSEA) for differentially expressed genes of RCNA3.

To elucidate RCAN3′s function, GO and KEGG pathway enrichment analyses were conducted. Biological processes primarily included calcineurin-NFAT signaling cascade, calcineurin-mediated signaling, and inositol phosphate-mediated signaling (Figure 10A). In cellular components, enrichment was observed in the protein serine/threonine phosphatase complex, and sarcolemma and cytoplasmic exosome. Molecular function enrichments featured protein serine/threonine phosphatase activity, phosphoprotein phosphatase activity, and cyclosporin A binding. KEGG enrichment analysis revealed involvement in T-cell receptor signaling, C-type lectin receptor signaling, Kaposi sarcoma-associated herpesvirus infection, osteoclast differentiation, and human T-cell leukemia virus 1 infection (Figure 10B). Key GO and KEGG categories are summarized in a network diagram (Figure 10C). Additionally, GSEA identified the top 10 significantly enriched pathways, primarily in PI3K signaling, insulin receptor signaling, and fibroblast growth factor receptor signaling (Figure 9D).

### Association with m6A ribonucleic acid methylation regulators in CESC

**Figure 11:**
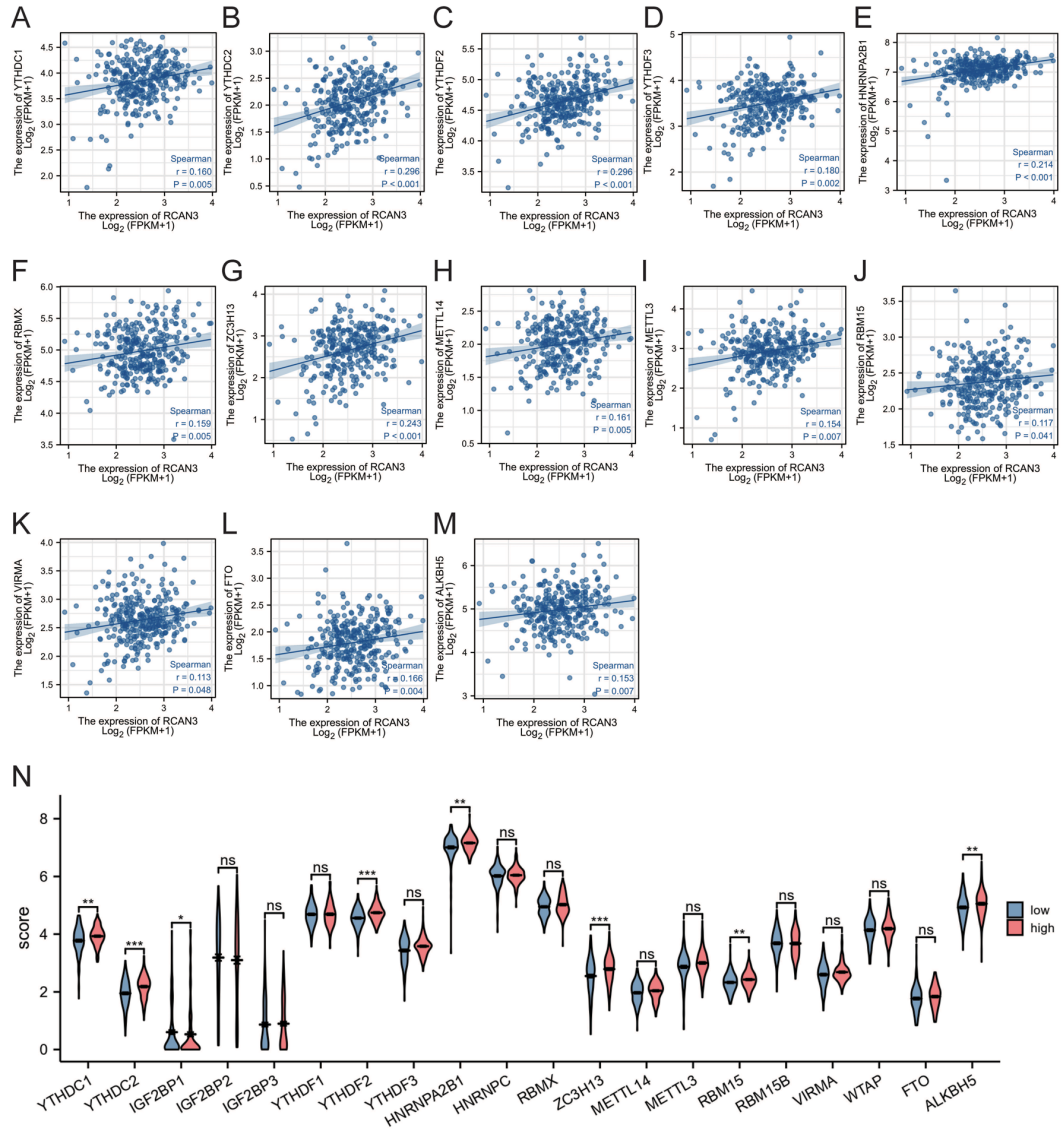
Associations between regulator of calcineurin 3 (RCAN3) expression and m6A-related genes in cervical squamous cell carcinoma and endocervical adenocarcinoma (CESC). (**A**) The correlation between RCAN3 and m6A related genes; (**B**) differential expression of m6A-related genes in groups with high and low RCAN3 expression in CESC tumor samples (*P < 0.05, **P < 0.01, ***P < 0.001).

Given the importance of m6A in CESC, the correlation between RCAN3 expression and m6A-related genes was investigated. RCAN3 expression was significantly positively correlated with 13 m6A-related genes in CESC, including *YTHDC1, YTHDC2, YTHDF2, YTHDF3, HNRNPA2B1, RBMX, ZC3H13, METTL14, METTL3, RBM15, VIRMA, FTO,* and *ALKBH5* (Figure 11A-M). The differential expression analysis between high-RCAN3 and low-RCAN3 expression groups in CESC indicated upregulated *YTHDC1, YTHDC2, IGF2BP1, YTHDF2, HNRNPA2B1, ZC3H13, RBM15,* and *ALKBH5* in the high RCAN3 group.

### Relationship with immune infiltration

**Figure 12:**
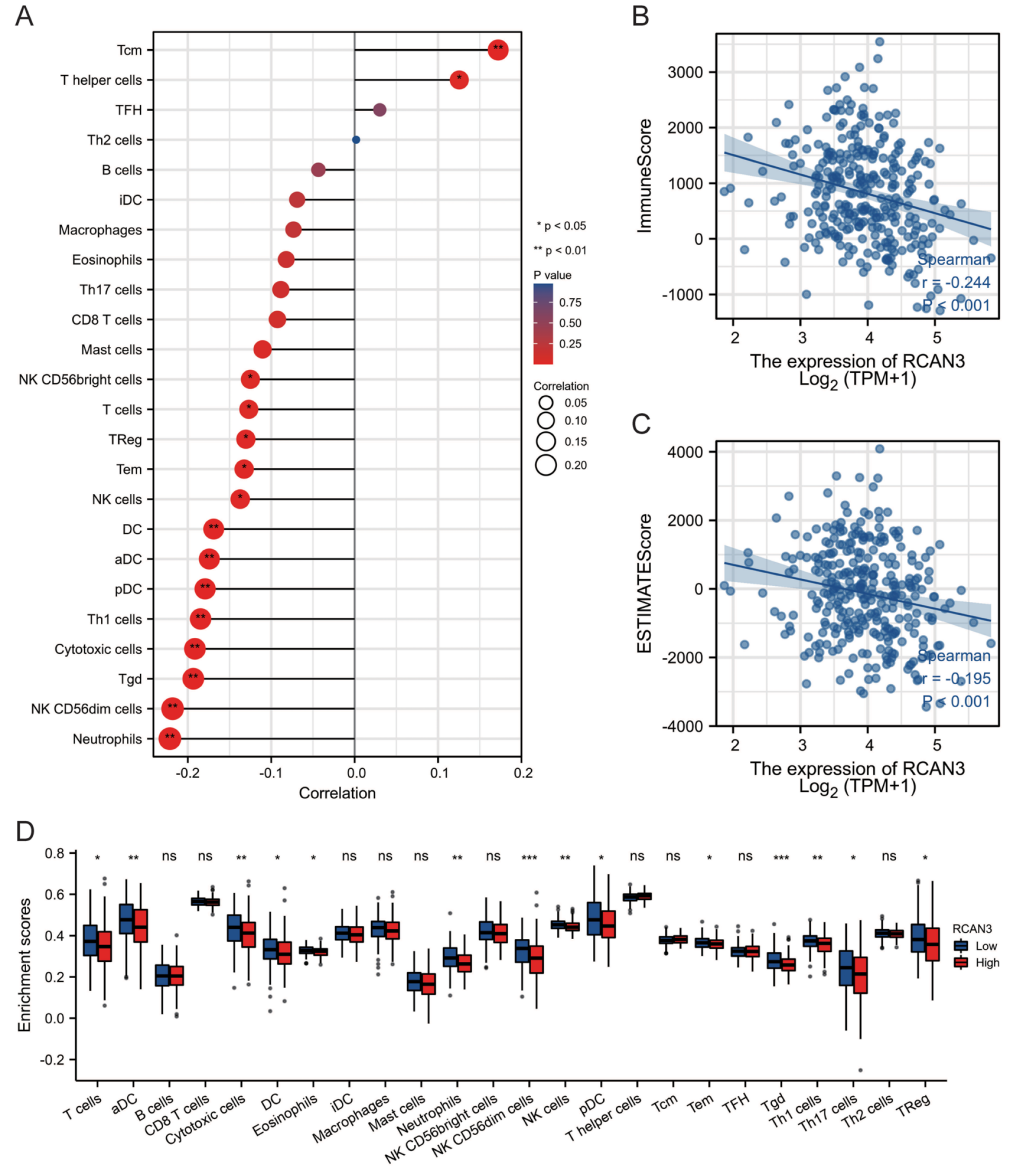
Associations between regulator of calcineurin 3 (RCAN3) expression and immune cell infiltration in cervical squamous cell carcinoma and endocervical adenocarcinoma (CESC). (**A**) The correlation between RCAN3 and immune cell infiltration; (**B,C**) the correlation between RCAN3 and immune score and estimate score; (**D**) differential expression of immune cells in the groups with high and low RCAN3 expression in CESC tumor samples (*P < 0.05, **P < 0.01, ***P < 0.001).

RCAN3 expression positively correlated with the expression of central memory cells (Tcm) and T helper cells but negatively correlated with the expression of NK CD56-bright cells, T cells, regulatory T cells (Treg), effector memory cells (Tem), NK cells, DC, aDC, pDC, Th1 cells, cytotoxic cells, gamma delta T cells (Tgd), NK CD56-dim cells, and neutrophils (Figure 12A). RCAN3 expression was significantly negatively correlated with the CESC microenvironment immune score and estimate score (Figure 12B, C). Additionally, immune cell infiltration differences between the high-RCAN3 and low-RCAN3 groups revealed a lower infiltration of T cells, aDC, cytotoxic cells, DC, eosinophils, neutrophils, NK CD56-dim cells, NK cells, pDC, Tem, Tgd, Th1 cells, Th17 cells, and Treg in the high-RCAN3 group (Figure 12D).

## DISCUSSION

To the best of our knowledge, the present study represents the first comprehensive exploration of RCAN3 expression and biological function across various cancers, with a specific focus on CESC. RCAN3 expression was increased across 25 cancer types and decreased in two cancer types. The differences in RCAN3 expression in different types of cancer reflect the complexity and diversity of cancer biology. They are also related to the unique molecular pathological features of each cancer, differences in signaling pathways, and the specificity of the tumor microenvironment. A decrease in RCAN3 expression in renal clear cell carcinoma and renal papillary cell carcinoma may be due to the presence of other compensatory mechanisms or alternative pathways that reduce RCAN3-dependent signal regulation. The upregulation of RCAN3 in other cancers may be related to enhanced cancer-related processes such as cell proliferation, migration, or inhibition of apoptosis. Additionally, RCAN3 expression significantly varied among immune subtypes in 6 cancer types and among molecular subtypes in 13 cancer types. Each immune subtype signifies a distinct immune microenvironment influencing patient survival across multiple cancer types (23).

RCAN3 demonstrated favorable diagnostic accuracy (AUC>0.7) in 20 cancer types and a notably high diagnostic accuracy (AUC>0.9) in 9 cancer types. Survival analysis showed a significant correlation between RCAN3 expression levels and OS, DSS, and PFI in CESC, LGG, and UVM. High RCAN3 expression was correlated with poorer OS, DSS, and PFI among CESC subgroups characterized by age, weight, histologic grade, histological type, and radiation therapy. Collectively, the present findings strongly affirm RCAN3′s potential as a diagnostic and prognostic biomarker, particularly in CESC.

The functional analysis focused on RCAN3-related genes pointed to a strong link with the CaN-NFAT signaling process, protein modification activities, specifically serine/threonine phosphatase actions, and the T cell receptor signaling mechanisms. Importantly, these findings match earlier research. They reinforce that RCAN3 connects with CaN via a consistent CIC sequence, which stops NFAT proteins in the cell's fluid from triggering gene activity (3,5,24). Calcineurin, a Ca^2+^/calmodulin-dependent serine/threonine phosphatase, plays a pivotal role in diverse cellular processes, including T-cell activation. Additionally, GSEA showed crucial effects on the PI3K signaling route and pathways connected to insulin. This includes how insulin receptors and insulin-like growth factor 1 receptors (IGF1Rs) pass signals. Insulin receptors and IGF1Rs are members of the same tyrosine kinase receptor family, and both exhibit substantial homology (25). IGF1Rs activation triggers multiple downstream signaling pathways, such as RAS/MEK/ERK and PI3K/Akt (26-28), which have been implicated in the development of various cancers, including cervical cancer (29). The present findings suggest that in CESC, RCAN3 may modulate its biological actions through these signaling pathways, paving the way for further biological investigations. Modification of m6A, the most prevalent mRNA alteration in mammals, is widely implicated in epigenetic regulation governing mRNA processing, translation, and stability (30,31). Mounting evidence underscores that m6A dysregulation is closely connected to cancer initiation and progression. For instance, METTL14 has been identified as an inhibitor of breast cancer cell proliferation, colony formation, and migration, while also diminishing the growth and self-renewal of glioblastoma stem cells (32,33). In contrast, METTL3 boosts cervical cancer cell proliferation by enhancing HK2 stability via YTHDF1 recruitment (34). This study sought to explore the potential link between RCAN3 expression and m6A modification in CESC. The findings unveiled a significant positive correlation between RCAN3 expression and *YTHDC1, YTHDC2, YTHDF2, YTHDF3, HNRNPA2B1, RBMX, ZC3H13, METTL14, METTL3, RBM15, VIRMA, FTO,* and *ALKBH5*. Moreover, the levels of *YTHDC1, YTHDC2, IGF2BP1, YTHDF2, HNRNPA2B1, ZC3H13, RBM15,* and *ALKBH5* were elevated in the RCAN3 high-expression group. The findings suggest that RCAN3 is subjected to m6A modification to regulate its translation and stability, which contributes to the development and progression of CESC. This presents an additional avenue for in-depth investigations into RCAN3 mechanism of action.

Accumulating evidence underscores the pivotal role of immune cell infiltration in tumor progression, recurrence, and responsiveness to immunotherapy. Previous investigations have established a link between increased immune cell infiltration and a favorable prognosis (35-37). For instance, Vincent et al, using the ESTIMATE algorithm to ascertain tumor purity, confirmed the absence of stromal and immune components in breast cancer (38). The present findings reveal a negative correlation between RCAN3 expression and the majority of immune cell types, as well as significant inverse correlations with immune scores within the CESC microenvironment. Furthermore, patients with CESC in the group with high RCAN3 expression exhibited diminished immune cell infiltration compared with their counterparts with low RCAN3 expression. The present data suggests that RCAN3 may facilitate immune evasion by tumor cells, leading to reduced immune cell infiltration in the CESC tumor microenvironment, ultimately promoting CESC tumorigenesis and progression.

RCAN3 expression is elevated in most tumor types and often leads to a poor cancer prognosis. This suggests that RCAN3 may be a promising target for cancer screening and diagnosis. For a targeted treatment of cancer patients, it is possible to design small-molecule drugs targeting the functional domain of proteins, reducing RCAN3, and thus improving cancer prognosis. In addition, RCAN3 test is expected to be used in combination with other clinical procedures and detection methods for the early screening and diagnosis of certain cancers. For example, RCAN3 testing of samples after cervical cancer cytological screening may help to improve the screening rate of cervical adenocarcinoma. In later-stage cancer, RCAN3 expression in different types of cervical adenocarcinoma can be studied for differential diagnosis.

This study has some limitations. The data sources, primarily TCGA and GTEx, may contain inherent biases and variations due to sample heterogeneity and different experimental techniques. The study’s reliance on bioinformatics analysis introduces sensitivity to data preprocessing and algorithm selection, and future studies should adopt multi-person data preprocessing methods or select multiple algorithms. The observational design of the study prevents us from concluding on causation. Some analyses involved multiple comparisons, potentially increasing the error rate. Smaller sample sizes in certain cancer types may have prevented us from detecting true effects or relationships. To address this issue, more data can be included in the database and new sources of data can be absorbed.

In conclusion, the present research reveals that RCAN3 could serve as a diagnostic and prognostic biomarker and therapeutic target for pan-cancer. Importantly, the study revealed the underlying mechanisms of RCAN3 in CESC, especially its close association with m6A modification and immune infiltration, which provides a bioinformatic basis for follow-up research. However, the present study was conducted through bioinformatics analysis, and the results require further validation.
